# Effect of Age and Height on the Chemical Properties of Muli Bamboo (*Melocanna baccifera*)

**DOI:** 10.1021/acsomega.2c05684

**Published:** 2022-10-22

**Authors:** Mohammad Jakir Hossain, Rupak Kumar Ghosh, Atanu Kumar Das, Roni Maryana, Shambhu Chandra Nath, Md. Rakibul Islam, Sujon Chandra Sarker

**Affiliations:** † Forest Chemistry Division, Bangladesh Forest Research Institute, Chittagong 4211, Bangladesh; ‡ Department of Forest Biomaterials and Technology, Swedish University of Agricultural Sciences, Umeå SE-90183, Sweden; § Research Center for Chemistry, National Research and Innovation Agency, Building 452 Kawasan PUSPIPTEK, South Tangerang, Banten 10340, Indonesia; ∥ Minor Forest Products Division, Bangladesh Forest Research Institute, Chittagong 4211, Bangladesh

## Abstract

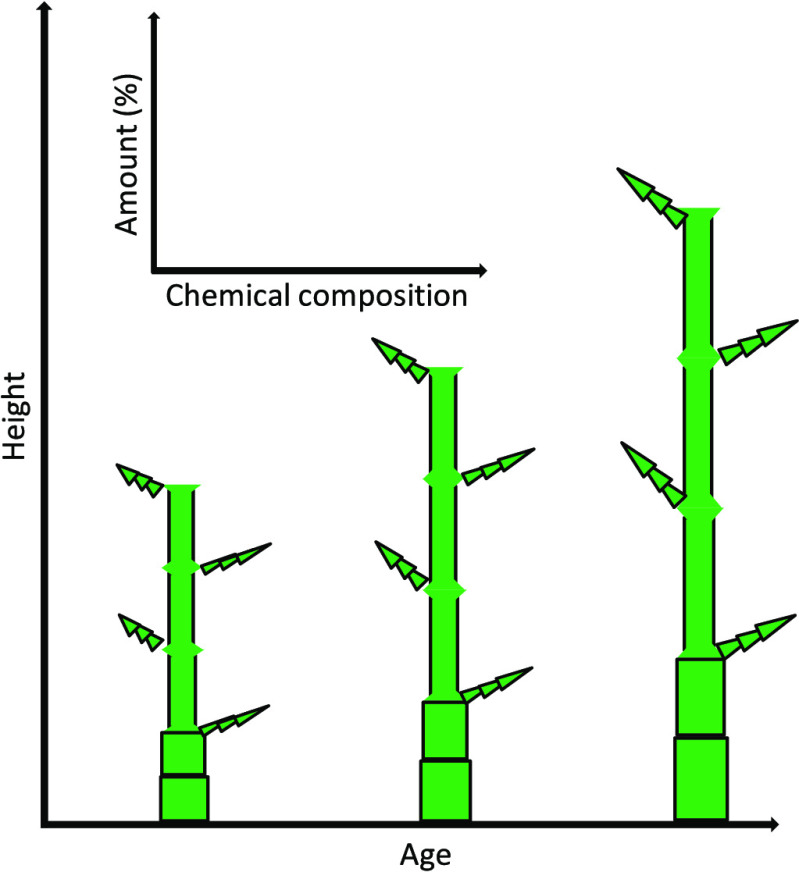

*Melocanna baccifera* is the most common bamboo species which grows naturally and gregariously covering large tracts of land in the forests of Chittagong Hill Tracts of Bangladesh. However, there is limited information about the chemical characterization of its culms for its utilization and processing. This paper aimed to determine the effect of age and height position on the chemical properties of *M. baccifera*. The highest value of holocellulose content was 74.66% for the top portion of 3-year-old bamboo, while the bottom part of 3-year-old bamboo showed the highest value of lignin (27.83%) and extractive (5.24%) content. For caustic soda (1% NaOH) solubility, the bottom portion of 1-year-old bamboo had shown the maximum value (25.67%), and it was the lowest (19.10%) for the top portion of 3-year-old bamboo. Ageing had a significant (*p* < 0.05) effect on all chemical properties, while the height position had a significant effect on the holocellulose and lignin content and water solubility. The chemical properties of *M. baccifera* can enable its proper utilization in the downstream process.

## 1 Introduction

Concerns over sustainability and environmental conservation related to the use of renewable resources have driven the development of a biobased economy that is devoted worldwide.^1^ In this context, the assertion of a substitute resource that mitigated the environmental problems has come into focus.^2^ The use of raw materials and products that are renewable, sustainable, and biocompatible has emerged as a study interest worldwide.^3^ Researchers have shown immense attention to developing wood^4−10^ and nonwood^11−24^-based products for society. In recent years, bamboo has attracted more attention as a renewable, cheap, fast-growing, and easily available material, and it is also compatible with existing processing technologies.^3^ Research has flourished rapidly due to its extensive availability and material characteristics comparable to wood.^25^ Recently, there has also been rising attention to the utilization of bamboo for pulp production,^26,27^ nanofiber extraction,^28^ composite materials,^25,29,30^ and biofuel production.^31,32^

Bamboo, a perennial woody grass with a high growth rate, wide range of applications, and renewability, occupies a noteworthy position in the 21st century as a versatile material.^33^ It attains maturity in 3 years, compared to wood, which takes more than 20 years.^34^ New shoots are generated from the harvested culm, as the root structure remains alive after harvest.^35^ It grows in plains and other nonproductive areas such as hilly and high-altitude mountainous regions and in most kinds of soils, except alkaline soils, deserts, and marsh.^36^ It is widely distributed in Asian countries and has become one of the most potential renewable nonwoody cellulosic materials because of its high productivity, rapid growth, and easy propagation.^28,31^

Muli bamboo (*Melocanna baccifera*) occurs naturally in Bangladesh, Myanmar, and northeastern India. It is occasionally planted in many botanical gardens in many Asian countries. It is the most common forest-grown bamboo species in Bangladesh with a small culm diameter and thin wall. This species constitutes 70–90% of the total bamboo forests of the country. The species can be easily recognized by the diffused clump habit, having culms that are 10–20 meters in height and 3–7 cm in diameter. The culms are strong and durable with inconspicuous nodes. These clumps are found to regenerate successfully even in heavily burnt and grazed areas and survive in unfavorable conditions of nature.^37^

Compared to other lignocellulosic biomass, bamboo has unique characteristics in chemical composition. Cellulose is the most abundant organic polymer on earth, making up 40–50% of the mass in bamboo.^38,39^ Hence, chemical characterization of bamboo is essential in determining its suitability for various applications. The accurate compositional analysis enables the evaluation of potential conversion yields and process economics.^40^ Knowledge of the physicochemical properties of bamboo is obligate for the efficient utilization of bamboo since information regarding basic properties is very limited. However, research is required to determine its diversified applications.

The properties of the plant are dependent on age and the height position.^41^ The amount of each chemical composition of bamboo varies with species, environmental condition, age, and location along with the culm height.^42^ The understanding of the variation in the chemical composition of bamboo with age and height difference is important for its potential utilization. Nevertheless, the chemical property variation of *M. baccifera* has not been reported yet. Hence, this study is conducted on a detailed analysis of chemical composition at different ages and heights of *M. baccifera*.

## 2 Materials and Methods

### 2.1 Raw Materials

Healthy, straight, and defect-free 1, 2, and 3-year-old bamboos were collected from Keucia Silviculture Research Station, Bangladesh Forest Research Institute (BFRI), Satkania, Chittagong (92°24′ E and 93°15′ E longitude and 24°22′ N and 25°8′ N latitude), Bangladesh. Reagent grade (≥95% purity) sodium hydroxide (NaOH), acetic acid (CH_3_COOH), sodium chlorite (NaClO_2_), and sulfuric acid (H_2_SO_4_) were received from Carolina Biological Supply Company, New York City, USA. Analytical grade (≥95% purity) benzene and ethanol were sourced from Merck KGA, Darmstadt, Germany.

### 2.2 Preparation of Raw Materials

Each of the culms was divided into three equal parts, that is, top, middle, and bottom. Then, the coded culms were dried and converted into strips. The bamboo strips of each portion were chipped and milled by a Hammer mill followed by drying in the sun. Then, the dried small particles were ground to produce fine powders by a Wiley mill. These powders were sieved to obtain 40–60 mesh size particles. The fine particles were stored in an air-tight container labeled with the appropriate code. The same procedure was applied to samples of all ages and heights.

### 2.3 Analysis of Chemical Properties

Chemical analysis was carried out based on the methods described elsewhere.^43,44^ In brief, the T-207 cm-99 standard was used to analyze cold and hot water solubility, and alkaline [1% caustic soda (NaOH)] solubility was determined by the T-212 om-02 standard. The extractive content was determined following the T-204 cm-97 standard. Holocellulose was analyzed by following the standard of T 249-75. Klason lignin was analyzed based on the T-222 cm-02 standard. The bamboo sample was analyzed at a minimum in triplicate for each type.

### 2.4 Data Analysis

The measured data obtained from the chemical analysis of the samples of various ages and heights were analyzed by SPSS (version 20). The normality and the significance of the main factors were analyzed using one-way analysis of variance (ANOVA) at a 5% significance level (*p* < 0.05). Mean separation was carried out using the least significant difference at *p* < 0.05.

## 3 Results and Discussion

### 3.1 Holocellulose

**Figure 1:**
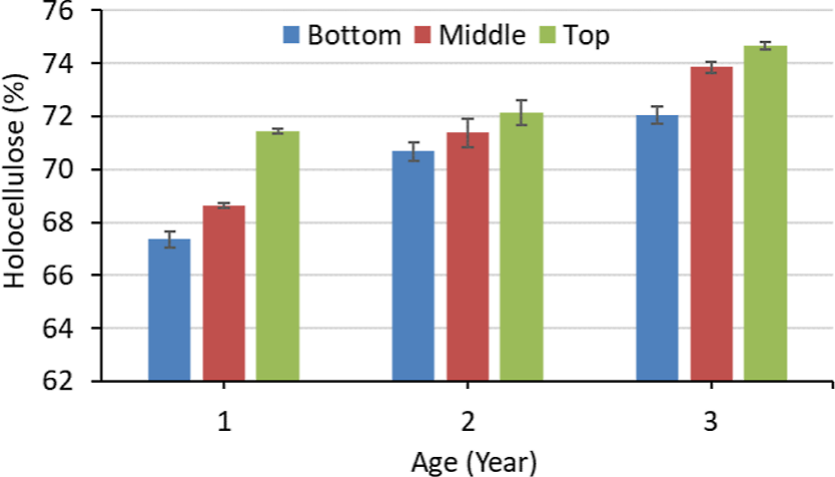
Holocellulose content of *M. baccifera* at different ages and heights.

The effect of age and height position on the holocellulose content of *M. baccifera* is presented in Figure 1. It increased with increasing height position and age. The highest holocellulose content was 74.66% for the top portion of 3-year-old *M. baccifera*. The difference between the top and bottom portions decreased for 2 and 3-year-old *M. baccifera*. The top portion showed 2.09 and 3.64% higher values of holocellulose content compared to the bottom portion for 2 and 3-year-old *M. baccifera*, respectively, while it was 6.06% for 1-year-old. The statistical analysis showed a significant effect (*p* < 0.05) of age and height position on the holocellulose content of *M. baccifera*. The top portion had the highest holocellulose content, and the bottom portion had the lowest holocellulose content. The lignin and extractive content increase during the ageing of bamboo.^45,46^ The authors have also observed the increment of holocellulose with aging. The formation of lignin and extractive may cause a lower degree of difference in holocellulose content of *M. baccifera* for 2 and 3-year-old *M. baccifera*. Li et al.^47^ have also observed a similar effect of age and height position on *Phyllostachys pubescens*. The presence of a higher vascular bundle at the top enhances the holocellulose content.^48^ Wood contains about 62–79% holocellulose,^49^ and this bamboo species showed the holocellulose content in the range of wood species. This bamboo can be used for pulp and paper, bioenergy, and biobased composite production. A 3-year-old bamboo can be a potential source of raw material since it contains the highest amount of holocellulose.

### 3.2 Lignin

**Figure 2:**
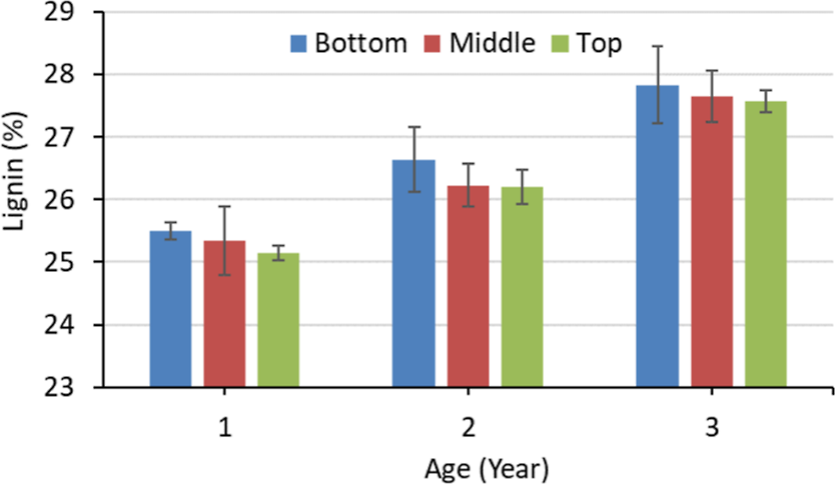
Klason lignin content of *M. baccifera* at different ages and heights.

Figure 2 presents the effect of age and height position on the Klason lignin content of *M. baccifera*. The bottom portion of the 3-year-old *M. baccifera* showed the highest lignin content (27.83%). The lignin content increased with ageing and the height position. The increment of lignin for the bottom, middle, and top was 4.5, 3.5, and 4.2%, respectively, from 1 to 2-year-old bamboo. It was 4.5, 5.4, and 5.2%, respectively when the bamboo became 3-year-old from 2-year-old. The accumulation of lignin was more in the middle and top when it turned to 3 years from 2 years. The top of 1-year-old had shown the lowest amount of lignin content (25.14%). According to statistical analysis, the effect of age and height on lignin content was significant (<0.5%). The top portion contains lots of new cells^50^ and consequently the top exhibits a lower lignin content. Wang et al.^48^ have reported that the height does not affect the Klason lignin content. Further studies may help to contribute to finding out the height effect on the lignin content of bamboo. Wood contains about 18–36% lignin,^49^ and the lignin content of *M. baccifera* was in the range of wood. The higher lignin content is problematic for the delignification. It is troublesome for the application in pulp and paper, bioenergy, and biobased composite. Therefore, less than 3-year-old bamboo is beneficial for use in the biorefinery process. However, lignin is also a potential source of biorefinery. On the other hand, 3-year-old bamboo contains the highest amount of holocellulose. Considering this, 3-year-old bamboo is a promising source of raw materials in the biorefinery process. The variation of lignin content should be considered for the delignification. A proper ratio of the height position can solve the problem. Further study with cost-benefit analysis can mitigate all the issues with appropriate applications.

### 3.3 Extractive

**Figure 3:**
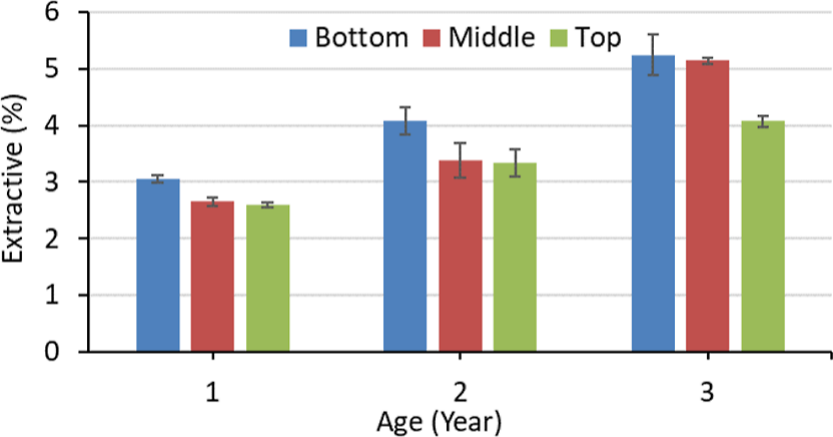
Extractive content of *M. baccifera* at different ages and heights.

From Figure 3, the variation in the extractive content can be seen according to age and the height position. The lowest extractive content was 2.59% for the top part of the 1-year-old, while the highest was 5.24% for the bottom part of the 3-year-old *M. baccifera*. The average percentage of extractive contents increases with the ageing of bamboo. The effect of age was significant (*p* < 0.05) on the extractive content. However, the height position showed an insignificant (*p* > 0.05) effect. Wang et al.^48^ have also observed that the height position does not have a strong influence on the extractive content. The presence of new cells may cause a lower amount of extractive content at the top. The higher extractive content is not beneficial to pulp and paper and bioenergy production. It hinders the delignification and further processing. Higher cellulose content is also important for pulp and paper and bioenergy production. On the other hand, higher extractive content protects from biodegradation, and it is beneficial to some biobased composites. Therefore, 3-year-old bamboo can be suitable for the biorefinery process. Further study can help to figure out its optimum utilization.

### 3.4 Solubility

#### 3.4.1 Water Solubility

**Figure 4:**
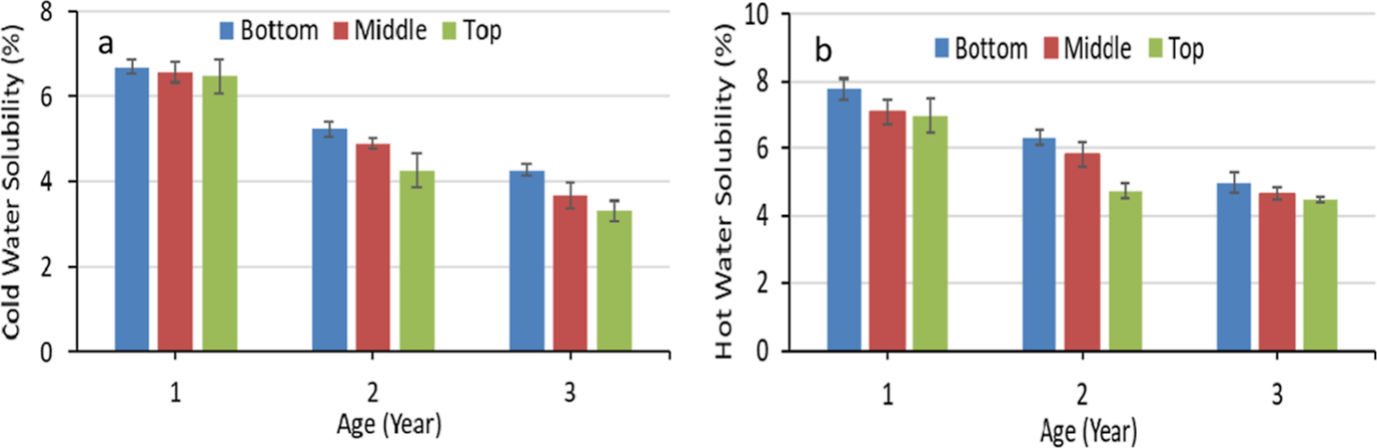
Cold (a) and hot (b) water solubility of *M. baccifera* at different ages and heights.

The cold and hot water solubility of *M. baccifera* at different ages and heights are presented in Figure 4. The hot water solubility was higher than the cold water solubility. The solubility of both types decreased with the increasing age and height. According to the statistical analysis, the effect of age and height on the cold and hot water solubility was significant (*p* < 0.05). The water solubility test indicates the levels of water-soluble extractives and sugars. The presence of fewer extractives according to the height position may result in low solubility. Furthermore, ageing may help to prevent water penetration forming more dead cells leading to less solubility. Azeez et al.^51^ have reported 4.70% of hot water solubility for *Bambusa vulgaris*. It is in the range of 3-year-old *M. baccifera* for the present study.

#### 3.4.2 Caustic Soda Solubility

**Figure 5:**
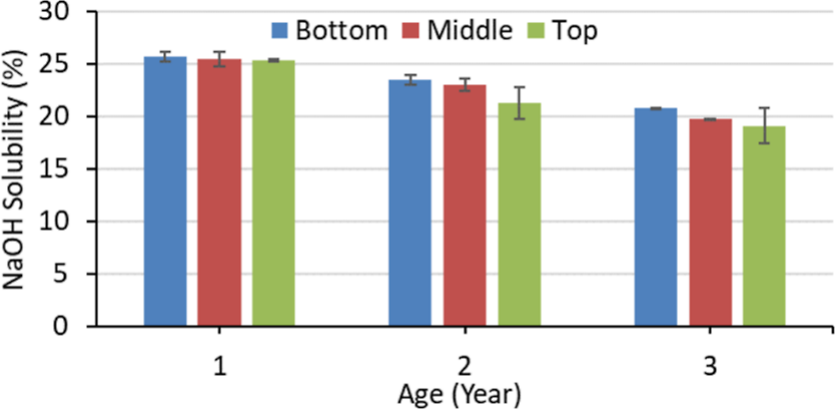
Caustic soda (1% NaOH) solubility of *M. baccifera* at different ages and heights.

The caustic soda (1% NaOH) solubility of *M. baccifera*, depending upon age and height, is presented in Figure 5. The bottom part of 1-year-old bamboo showed the highest (25.67%) NaOH solubility, and the top portion of the 3-year-old bamboo showed the lowest (19.10%). Ageing had a significant (*p* < 0.05) effect on NaOH solubility, and it decreased with the ageing of bamboo. The effect of height position on NaOH solubility was insignificant (*p* > 0.05) for this study. The extractive and lignin content increase with the ageing of bamboo.^47^*M. baccifera* had shown a similar trend in this study. This might prevent the solubility of low molecular weight carbohydrates in a 1% NaOH solution.

## 4 Conclusions

The variation of chemical properties of *M. baccifera* with age and height position was investigated in this study. It was found that there was a significant effect of age on the chemical properties. However, the height position had shown a significant effect on the holocellulose and lignin content and water solubility. The amount of holocellulose and lignin content was in the range of wood and other bamboo species. Considering these, *M. baccifera* can be a potential source of biorefinery. Further studies on the effect of age in the downstream process, that is, pulping, bioethanol, and so forth, may help to figure out its potential applications.
